# Association of dietary saturated fatty acid intake with depression: mediating effects of the dietary inflammation index

**DOI:** 10.3389/fnut.2024.1396029

**Published:** 2024-06-14

**Authors:** Caijuan Qi, Ruoyu Gou

**Affiliations:** ^1^Zhenyuan County Center for Disease Control and Prevention, Qingyang, Gansu, China; ^2^School of Public Health, Ningxia Medical University, Yinchuan, Ningxia, China

**Keywords:** SFAs, DII, NHANES, depression, mediation

## Abstract

**Background:**

Diet and dietary inflammation play an important role in depression. The aim of this study was to assess the association of SFAs with depression risk and the mediating role of DII.

**Method:**

Among 22, 478 U.S. adults (≥ 20, years old) according to the National Health and Nutrition Examination Survey (NHANES), univariate logistic regression, and multivariate logistic regression were used to evaluate the association between dietary intake of SFAs and the risk of depression. Dietary inflammation levels were evaluated using the DII. Mediation analysis was used to investigate the risk of DII and depression. The nonlinear relationship between SFAs and depression was assessed using restricted cubic spline (RCS).

**Results:**

There was a significant difference in SFA 6.0 dietary intake between depression and non-depression individuals. After adjusting for potential confounders, multifactorial logistic regression results showed that SFA 8.0 (Q3 1.58 (1.09, 2.30), *p*-value = 0.017; Q4 1.55 (1.00, 2.42), *p*-value = 0.050) may increase the prevalence factor for depression, SFA 14.0 (Q3 0.67 (0.47, 0.94), *p*-value = 0.020) may decrease the risk of depression. There were sex and age differences in the effects of different subtypes of SFAs on depression. Dietary intake of SFA 12.0 content showed a nonlinear relationship with the risk of depression (*p*-value = 0.005). Furthermore, DII was recognized as a mediator of the association between SFAs and the risk of depression.

**Conclusion:**

The findings suggest that dietary intake of SFAs is associated with the risk of depression in relation to the chain length of SFAs, and this may be due to the mediating effect of DII.

## 1 Introduction

Depression, a prevalent mental illness, can manifest in various ways. Symptoms of a depressive episode may include feelings of sadness, irritability, or emptiness, difficulty concentrating, overwhelming guilt or low self-esteem, a sense of hopelessness, and suicidal ideation. This condition is a significant contributor to disability worldwide (1, 2). Globally, an estimated 3.8% of the population suffers from depression, including 5% of the adult population (4% of men and 6% of women, and women are more likely to suffer from depression than men), and 5.7% of adults (≥ 60 years old), and it is significant to highlight that during the COVID-19 crisis, there was a surge in cases by 50 million worldwide (1, 3). The pathogenic mechanisms of depression are complex and have not been clearly reported to date. Although genetic factors have received much attention, the environment plays a decisive role in pathogenesis. Such as dietary nutritional intake, maltreatment, chronic stress, and disability are the most relevant environmental and social factors (4, 5).

Various nutrients play a crucial role in the onset and management of depression. Deficiencies in calcium, magnesium, iron, zinc, vitamin D, B vitamins, and saturated fatty acids have been linked to an increased risk of depression (6–8). The research is shedding light on the impact of lipid consumption on brain function (9). Lipids, a group of organic compounds that include fatty acids, can be influenced by dietary choices, leading to changes in brain lipid composition. Consumption of a diet rich in saturated fatty acids (SFAs) has been associated with improved mood and decreased irritability compared to a low-fat diet (10). A recent study suggests that saturated fatty acid (SFA) intake is not associated with depressive symptoms, and that it is monounsaturated fatty acids (MUFA) and polyunsaturated fatty acids (PUFA) that are important factors in alleviating depression (11). Additionally, it has been reported that increasing 22:0, 23:0, and 24:0 may be protective against the risk of depression (12). SFAs can be categorized based on carbon chain length: short-chain SFAs (2–6 carbons), medium-chain SFAs (8–12 carbons), and long-chain SFAs (≥ 14 carbons). Moreover, brief-chain SFAs and medium-chain SFAs offer protection against depression (6). The study discovered that the intake of SFAs was a predictor of depressive symptoms in women of middle age. The severity of their symptoms was directly related to their consumption of SFAs (13, 14), while the total intake of SFAs, such as SFA 14.0, 16.0, and 18.0, was linked to a higher likelihood of experiencing depressive symptoms (6). The absorption and metabolism effects of SFAs differ based on the length of their carbon chains, and the discrepancies in findings among these studies may be due to the predominant types of SFAs consumed in the diet (15). Additionally, the exact mechanism by which SFAs influence depression remains mostly unclear. The DII evaluates the combined impact of all inflammatory and anti-inflammatory dietary elements (16). Studies suggest that individuals with higher DII scores tend to consume more SFAs (17). Research has indicated that diets high in SFAs can promote neuroinflammation and raise the likelihood of depression (18, 19). Increased consumption of SFAs can activate astrocytes, trigger the release of pro-inflammatory cytokines, raise levels of CRP, and induce neuroinflammation, thereby heightening the risk of depression (20–22). Consequently, our hypothesis is that a diet rich in SFAs might heighten the risk of depression and that the DII could play a mediating role.

We conducted a continuous cross-sectional study to ex amine the relationship between SFAs and the risk of depression using data from the 2005–2020 NHANES. Additionally, we assessed levels of dietary inflammation using the DII and investigated the potential mediating effect of DII.

## 2 Methods

### 2.1 Study populations

NHANES is a continuing survey of the health and nutritional status of the United States population, and it is conducted by members of the Centers for Disease Control and Prevention (NCHS). Participant data are obtained via an intricate, classified, multistage probabilistic cluster sampling design, and involves 5,000 people each year. The collected information includes subject demographics, body measurements, laboratory test results, and dietary information. Prior to the survey, all subjects provided a written informed consent for survey participation and data usage in health-related statistical research. Program details, data acquisition protocols, and available data are open to the public, and can be accessed at http://www.cdc.gov/nchs/nhanes.html.

**Figure 1:**
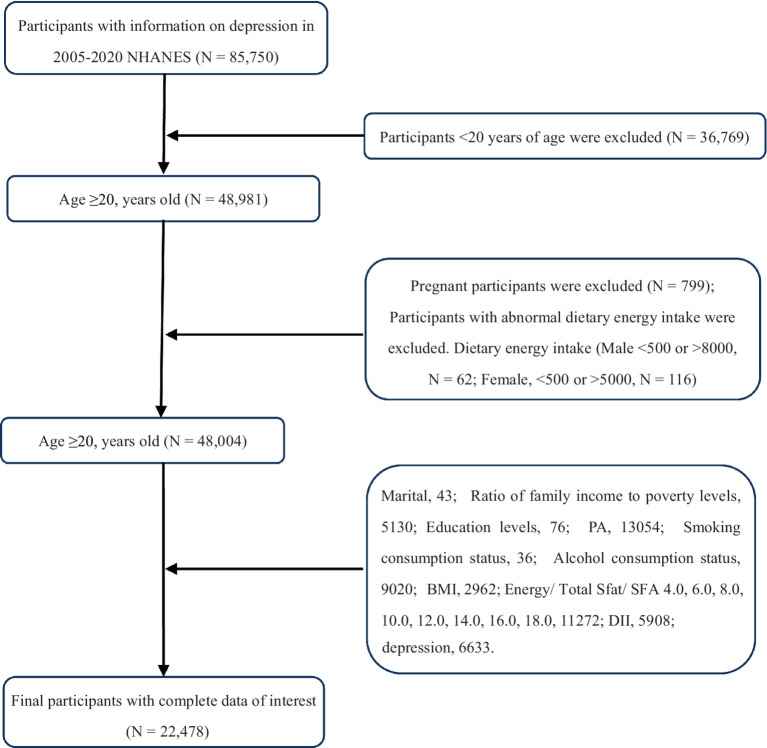
Flow chart of participants selection. NHANES, National Health and Nutrition Examination Survey.

We examined 8 survey cycles spanning the years 2005–2019. Subjects were aged ≥20 with complete data. Ultimately, we included 22,478 subjects in the analysis, and after estimating the weights for the complex sample, the results of this study reflect the health risks of 125,025,991 participants, as shown in Figure 1.

### 2.2 Assessment of SFAs

Dietary sources of energy, Total SFA, and their subtypes (SFA 4.0, 6.0, 8.0, 10.0, 12.0, 14.0, 16.0, 18.0) were collected through two 24-h recall interviews. Nutrient types were chosen and computed based on NHANES support material.^1^ The method used to measure dietary intake was thoroughly explained in the measurement guide provided by the CDC.^2^ The calculations for dietary nutrient intakes were done using the Food and Nutrition Database for Dietary Studies 5.0 standard set forth by the Ministry of Agriculture (23, 24).

### 2.3 Assessment of depression

NHANES provided a tool for measuring depression known as the PHQ-9 questionnaire, which assesses the presence of nine signs and symptoms of depression based on the Diagnostic and Statistical Manual of Mental Disorders-IV (DSM-IV) criteria. Responses to the questionnaire are rated on a scale from “Not at all” to “Nearly every day,” with each response corresponding to a score of 0 to 3. The total score is calculated to be between 0 and 27 points. In the context of this study, depression was defined as a total PHQ-9 score equal to or greater than 10 (25).

### 2.4 Measurement of dietary inflammatory index (DII)

DII was initially introduced in 2004, with its first-generation protocols and verification being outlined in 2009. An enhanced version of DII was later presented in 2014, incorporating data from 11 national food and nutrition databases across 4 continents. In addition to analyzing food parameters, the study also measured inflammatory markers. These markers were used to calculate food parameter-specific DII scores, which were then used to determine an overall DII score for each individual included in the analysis (26). The NHANES data comes with 28 available parameters that can be used to compute DII. These include energy, protein, carbohydrate, dietary fiber, total fatty acid, total saturated fatty acid, monounsaturated fatty acids (MUFA), polyunsaturated fatty acids (PUFA), cholesterol, β-carotene, niacin, folate, magnesium, iron, zinc, selenium, caffeine, alcohol, n3-PUFA, n6-PUFA, and vitamins A, B1, B2, B6, B12, C, D, and E. An augmented DII score represents an inflammation-triggering diets, and a reduced score represents an anti-inflammatory diet (27).

### 2.5 Defining covariates

Patient demographics, including age, sex, race/ethnicity, marital status, income level, education level, BMI, smoking habits, alcohol consumption, and physical activity (PA), were considered as covariates. Race was categorized into white, black, Mexican, or other. Education level was reported as less than 11th grade, high school graduate, or college graduate and higher. Income level was divided into four groups based on a scale ranging from 1.3 to greater than 5. A higher ratio indicated a higher income level. Marital status options included married, divorced, or unmarried. Alcohol consumption patterns and sex of consumers were noted, with consumption levels categorized as never, former, mild, moderate, or heavy. Smoking habits were classified as current, former, or never smokers. Non-smokers were individuals who had smoked less than 100 cigarettes in their lifetime, ex-smokers had smoked more than 100 cigarettes but had quit, and current smokers were individuals who had smoked over 100 cigarettes with varying levels of consistency. BMI categories were defined as less than 25 kg/m^2^, 25–30 kg/m^2^, or greater than 30 kg/m^2^.

### 2.6 Statistical analysis

We employed the NHANES criteria for statistical analysis (oversampling, stratification, and clustering) to predict the appropriate number of U.S. adults to use in this study. In addition, we also evaluated the required statistical tests for weight adjustment. Categorical variables are presented in n (%), and inter-group differences were assessed via chi-squared test. Spearman correlation analysis was used and correlation coefficients were reported.

Nutrient intake was corrected using the residual method. Nutrient intakes were grouped into quartiles (Q1, Q2, Q3, Q4). The lowest quartile (Q1, first quartile) was defined as the reference group in each model. We employed logistic regression models to assess relationships between SFAs intake with depression. We also employed linear regression models to assess relationships between SFAs with DII and DII with depression. The acquired data are presented as weighted (OR [95% CI]). We also performed subgroup analyses of sex and age of patients as well as interactions to further elucidate the above relationships in different populations.

We generated RCS plots to display patterns in variables of significance in logistic regression. Using the RCS plots, we determined the presence or absence of a nonlinear association between the mentioned exposure factors and depression. The possibility of the DII as a modulator of the SFAs intake and depression association was further examined using a parallel mediation model employing a quasi-Bayesian Monte Carlo technique with 1,000 normal approximation-based simulations (R package “mediation”). Direct effect (DE) indicated the dietary saturated fatty acids and their subtype intake-mediated regulation of depression in the absence of mediators. Indirect effect (IE) indicated the SFAs intake-mediated modulation of depression using a mediator. Mediation was quantified as follows: IE divided by TE (total effect).

All remaining statistical analyses were performed using R software (version 4.2.2)^3^; the following packages were used: nhanesR (version 0.9.2.8), survey, dplyr, tidyverse, do, finalfit, Formula, rms, and foreign. The statistical tests were two-sided, and the results were considered statistically significant when the *p*-value < 0.05.

## 3 Result

### 3.1 Characteristics of the study population and SFAs distribution

**Figure 2:**
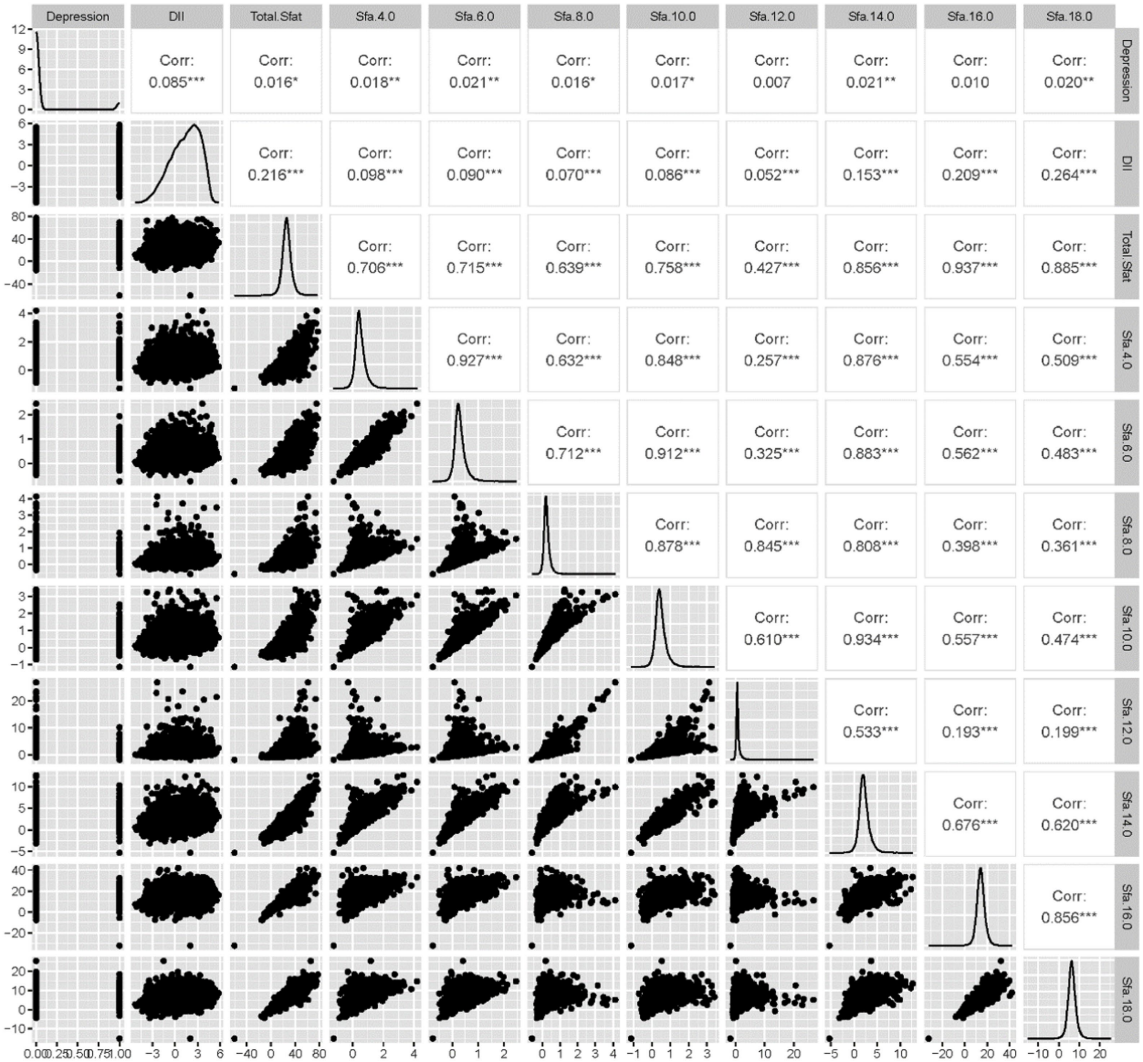
Matrix diagram of correlation between SFAs and depression.

Among the 22,478 adults surveyed, 1,684 participants were found to have depression. The study revealed that SFA6.0, SFA8.0, Energy, DII, Age, Sex, Ethnic/race, Marital status, Ratio of family income to poverty levels, Education levels, PA, Smoking consumption status, Alcohol consumption status, and BMI were statistically significant factors among participants with depression compared to those without. Conversely, factors such as Total SFAs, 4.0, 10.0, 12.0, 14.0, 16.0, and 18.0 did not show statistical significance between the two groups. The correlation analysis indicated that SFAs were generally correlated with DII and depression, with the exception of SFA16.0. Further details can be seen in Table 1 and Figure 2.

**Table 1:** Characteristics of the study population and SFAs distribution.

Parameter	No. of participants (Weighted %); (mean (SE))			
	All participants (*N* = 22, 478)	Non-depression (*N* = 20, 794)	Depression (*N* = 1, 684)	*p*-value^a^
Total SFA	0.54 (0.08)	0.51 (0.09)	0.96 (0.30)	0.171
SFA 4.0	0.04 (0.00)	0.04 (0.00)	0.06 (0.01)	0.243
SFA 6.0	0.02 (0.00)	0.02 (0.00)	0.04 (0.01)	0.029
SFA 8.0	0.01 (0.00)	0.01 (0.00)	0.03 (0.01)	0.065
SFA 10.0	0.03 (0.00)	0.03 (0.00)	0.05 (0.01)	0.138
SFA 12.0	0.03 (0.01)	0.03 (0.01)	0.05 (0.03)	0.616
SFA 14.0	0.11 (0.01)	0.10 (0.01)	0.19 (0.04)	0.086
SFA 16.0	0.14 (0.04)	0.13 (0.04)	0.33 (0.14)	0.197
SFA 18.0	0.11 (0.02)	0.10 (0.02)	0.21 (0.08)	0.202
Energy	2147.28 (7.99)	2153.90 (8.33)	2051.11 (25.70)	< 0.001
DII	1.27 (0.03)	1.22 (0.03)	1.93 (0.06)	< 0.001
Age				
20–44	10,237 (47.39)	9,432 (47.17)	805 (50.50)	< 0.001
45–64	7,858 (36.97)	7,189 (36.81)	669 (39.32)	
≥ 65	4,383 (15.64)	4,173 (16.01)	210 (10.18)	
Sex				
Female	10,811 (49.01)	9,800 (48.28)	1,011 (59.48)	< 0.001
Male	11,667 (50.99)	10,994 (51.72)	673 (40.52)	
Ethnic/race				
White people	10,309 (71.69)	9,584 (72.11)	725 (65.62)	
Black people	4,724 (9.71)	4,352 (9.54)	372 (12.13)	< 0.001
Mexican	3,005 (7.05)	2,783 (7.03)	222 (7.22)	
Other	4,440 (11.56)	4,075 (11.32)	365 (15.03)	
Marital				
Married	13,721 (65.22)	12,950 (66.42)	771 (47.80)	< 0.001
Never married	4,381 (18.41)	3,977 (17.95)	404 (25.12)	
Separated	4,376 (16.37)	3,867 (15.63)	509 (27.07)	
Ratio of family income to poverty levels				
< 1.3	6,085 (17.47)	5,271 (16.13)	814 (36.88)	< 0.001
1.3–3	6,887 (26.97)	6,374 (26.71)	513 (30.69)	
`3–5	4,783 (25.65)	4,567 (26.15)	216 (18.34)	
≥ 5	4,723 (29.91)	4,582 (31.00)	141 (14.09)	
Education levels				
Less than 11th grade	3,960 (11.15)	3,480 (10.56)	480 (19.68)	< 0.001
High school graduate	11,246 (56.35)	10,606 (57.24)	640 (43.54)	
College graduate or above	7,272 (32.50)	6,708 (32.20)	564 (36.78)	
PA				
< 600, Mets, mins/week	5,103 (22.10)	4,673 (21.89)	430 (25.29)	0.010
≥ 600, Mets, mins/week	17,375 (77.90)	16,121 (78.11)	1,254 (74.71)	
Smoking consumption status				
Never	5,501 (25.40)	5,129 (25.61)	372 (22.39)	< 0.001
Former	12,533 (56.08)	11,864 (57.32)	669 (38.13)	
Now	4,444 (18.52)	3,801 (17.07)	643 (39.47)	
Alcohol consumption status				
Never	2,758 (10.47)	2,483 (10.21)	275 (14.26)	< 0.001
Former	4,737 (21.71)	4,241 (21.15)	496 (29.85)	
Mild	8,500 (39.79)	8,055 (40.61)	445 (27.79)	
Moderate	3,946 (19.15)	3,626 (18.98)	320 (21.72)	
Heavy	2,537 (8.88)	2,389 (9.05)	148 (6.38)	
BMI				
< 25, kg/m^2^	6,539 (30.66)	6,121 (30.80)	418 (28.53)	< 0.001
25–30, kg/m^2^	7,440 (33.23)	6,998 (33.75)	442 (25.71)	
≥ 30, kg/m^2^	8,499 (36.11)	7,675 (35.45)	824 (45.76)	

a T-test for continuous variables and Rao-Scott χ^2^test for categorical variables.

### 3.2 Univariate logistic regression results of SFAs and depression

**Figure 3:**
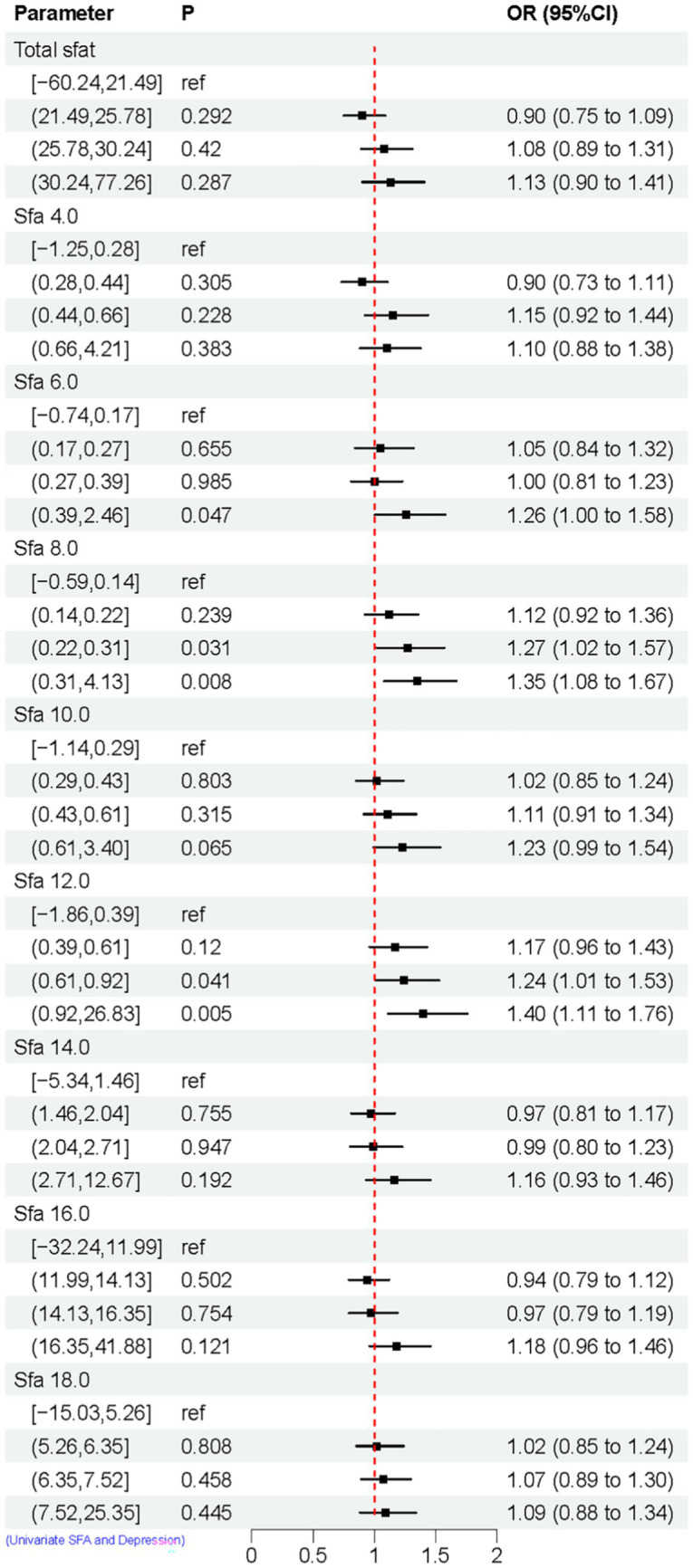
Univariate logistic regression results of SFAs and depression. Model Adjusted for sex, age, ethnic/race, marital, family income-to-poverty ratio, education levels, PA, BMI, alcohol consumption status and smoke consumption status, DII.

The relationship between categorized SFAs and depression risk was assessed using weighted univariate logistic regression models. Compared to the control group, the results indicated that SFA 6.0 [Q4, 1.26 (1.00, 1.58)], SFA 6.0 [Q4, 1.26 (1.00, 1.58)], and SFA 8.0 [Q3, 1.27 (1.02, 1.57)], [Q4, 1.35 (1.08, 1.67)], SFA 12.0 [Q3, 1.24 (1.01, 1.53)], [Q4, 1.40 (1.11, 1.76)]. These findings suggest that SFA 6.0, 8.0, and 12.0 might elevate the risk of depression, as shown in Figure 3.

### 3.3 Multivariate logistic regression results SFAs and depression

**Figure 4:**
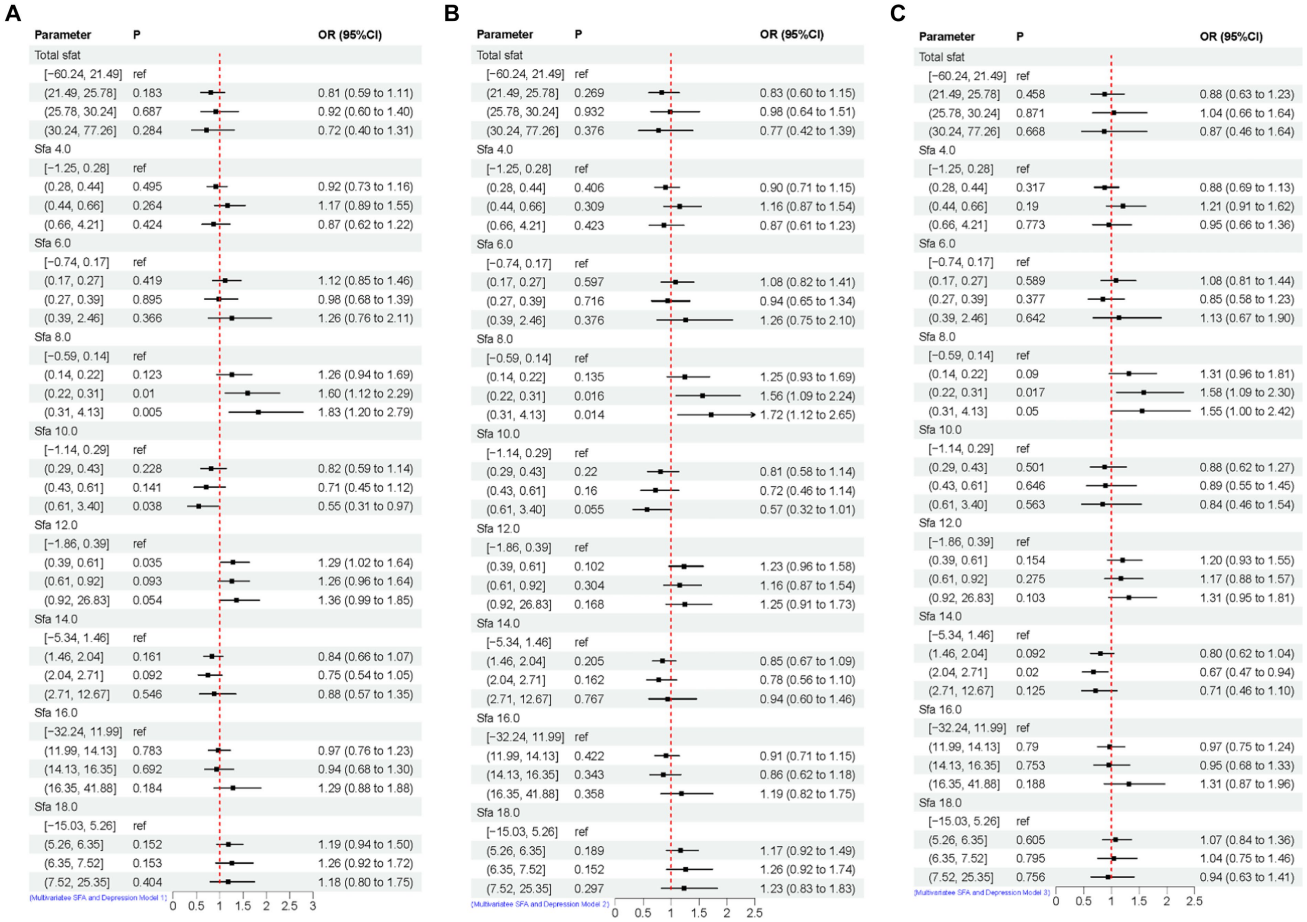
Multivariate logistic regression results of SFAs and depression. Model 1 **(A)**, no adjustment for any potential influence factors. Model 2 **(B)**, adjusted for sex, age and ethnic/race. Model 3 **(C)**, adjusted for sex, age, ethnic/race, marital, family income-to-poverty ratio, education levels, PA, BMI, alcohol consumption status and smoke consumption status, DII.

**Figure 5:**
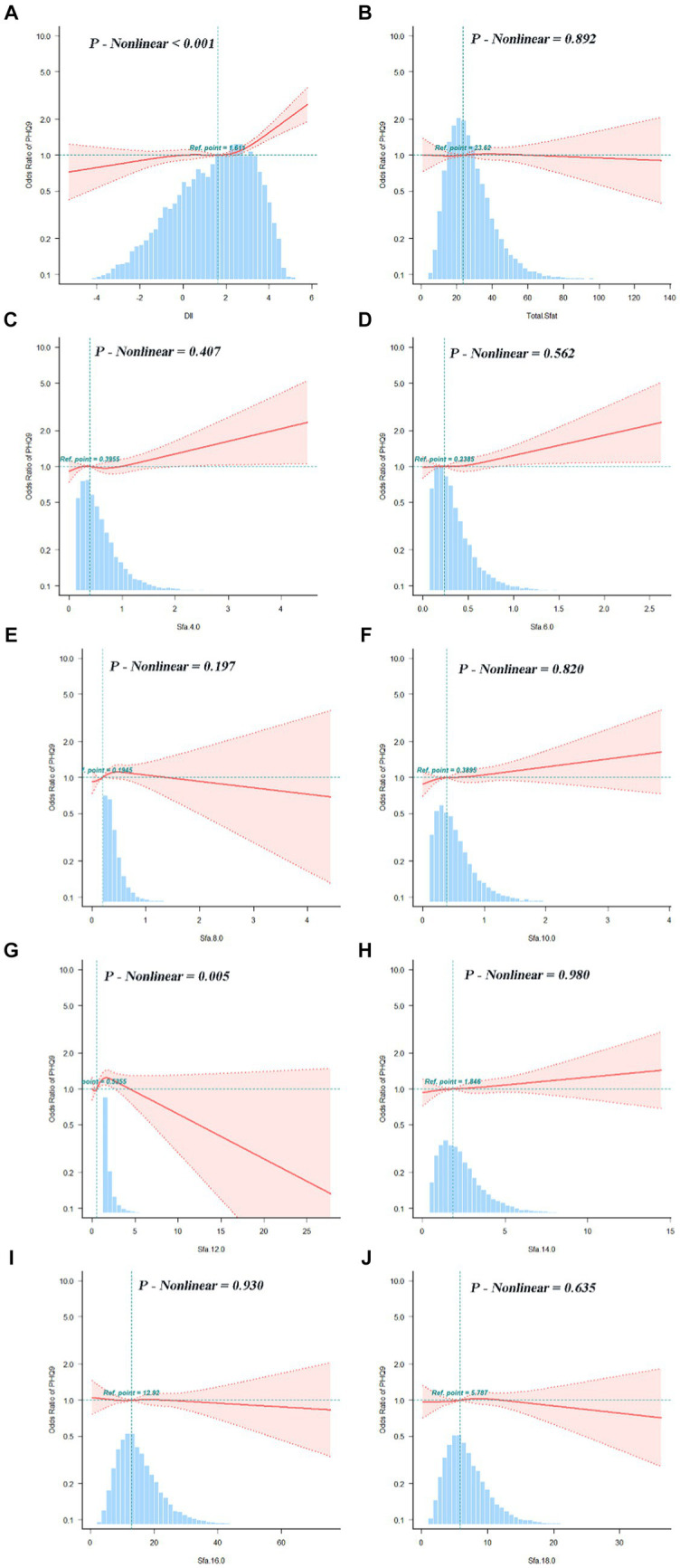
Dose–response relationships between DII **(A)**, SFAs and their subtypes **(B–J)** and depression. OR (95% CI) (shaded areas) were adjusted for sex, age, ethnic/race, marital, family income-to-poverty ratio, education levels, PA, BMI, alcohol consumption status and smoking consumption status, DII. Ref indicate the minimal threshold for the beneficial association with estimated OR = 1. OR, odds ratio.

The association between adjusted SFAs (continuous variables were grouped into quartiles) and depression was demonstrated by weighted multivariate logistic regression models, adjusting for multiple latent variables. In Model 1, Model 2, and Model 3, the results remained consistent at dietary intakes of Q3 and Q4 for SFA 8.0 compared with controls, both of which are likely to be associated with an increased risk of developing depression. OR [95% CI] was [Q3, 1.60 (1.12, 2.29), 1.56 (1.09, 2.24), 1.58 (1.09, 2.30)], [Q4, 1.83 (1.20, 2.79), 1.72 (1.12, 2.65), 1.55 (1.00, 2.42)]. In Model 3, SFA 14.0 is [Q3, 0.67 (0.47, 0.94)] compared to the control group, as shown in Figure 4. A nonlinear relationship was observed between DII and depression [*P-nonlinear* < 0.001; threshold value: 1.611 (OR = 1)]. A nonlinear relationship was observed between SFA 12.0 and depression [*P-nonlinear* = 0.005; threshold value: 0.536 (OR = 1)], as shown in Figure 5.

### 3.4 Linear regression results of SFAs with DII and DII with depression

**Figure 6:**
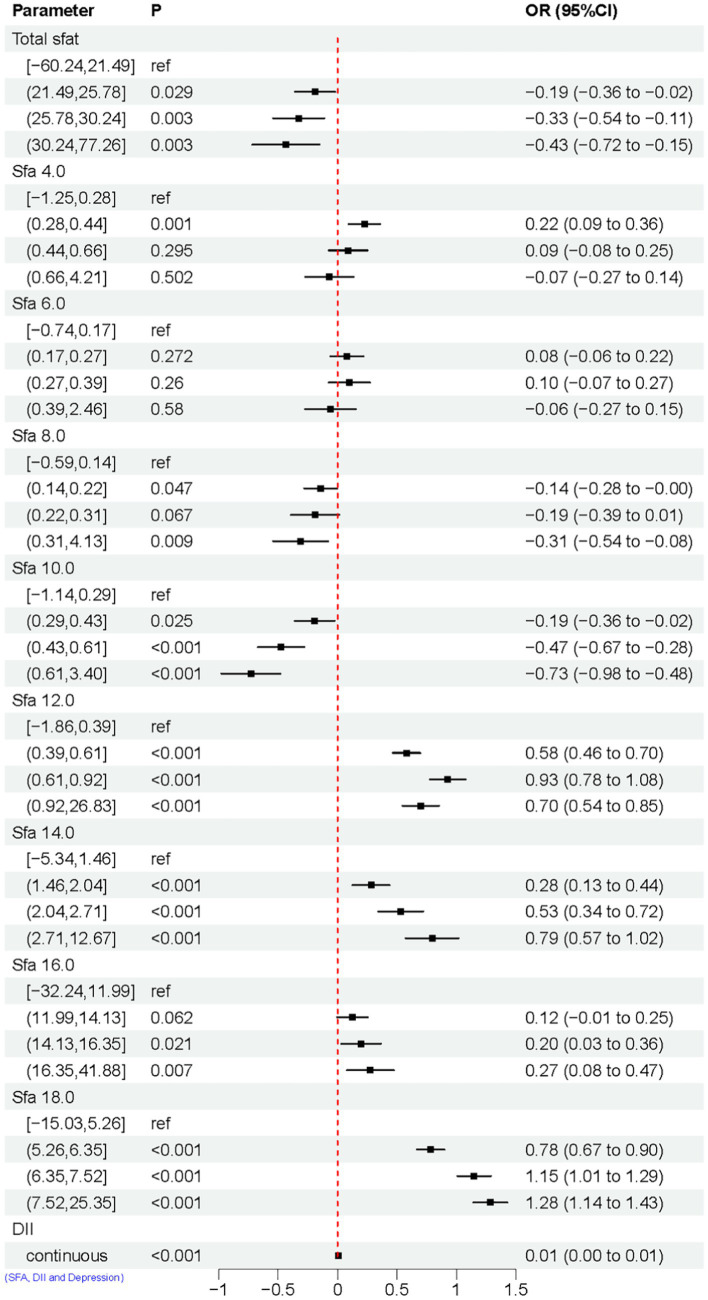
Linear regression results of SFAs with DII and DII with depression. Model adjusted for sex, age, ethnic/race, marital, family income-to-poverty ratio, education levels, PA, BMI, alcohol consumption status and smoke consumption status, DII.

The study illustrates the relationship between SFAs and DII through linear regression analysis. Total SFAs, 8.0, and 10.0 show a negative correlation with DII, while SFA 4.0, 12.0, 14.0, 16.0, and 18.0 are linked to an increase in DII. Furthermore, the linear regression analysis also highlights the connection between DII and depression, revealing that DII is associated with a higher risk of depression, as shown in Figure 6.

### 3.5 Mediation analysis

**Figure 7:**
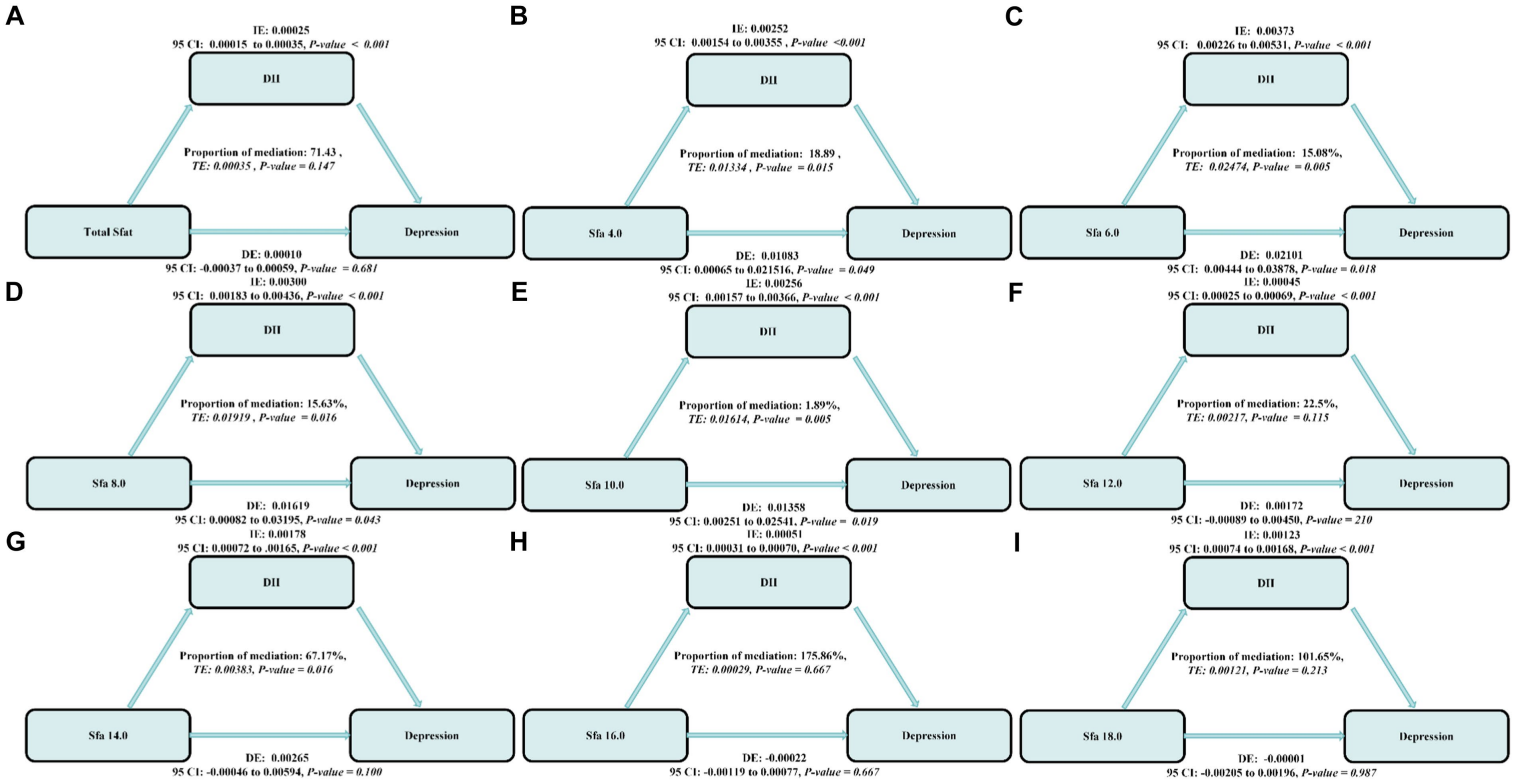
The estimated proportions of the associations between SFAs and their subtypes **(A–H)** and depression mediated effect by dietary inflammatory index **(I)**. Model adjusted for sex, age, ethnic/race, marital, family income-to-poverty ratio, education levels, PA, BMI, alcohol consumption status and smoking consumption status, DII. IE, the estimate of the indirect effect; DE, the estimate of the direct effect; Proportion of mediation = IE/DE + IE, OR, odds ratio.

The mediating role of DII was prevalent in the association of SFAs with depression. The mediation ratios were, respectively, the Total SFAs (mediation proportions: 71.43%, *p*-value ≤ 0.001),SFA 4.0 (mediation proportions: 18.89%, *p*-value ≤ 0.001), SFA 6.0 (mediation proportions: 15.08%, *p*-value ≤ 0.001),SFA 8.0 (mediation proportions: 15.63%, *p*-value ≤ 0.001), SFA 10.0 (mediation proportions: 1.89%, *p*-value ≤ 0.001), SFA 12.0 (mediation proportions: 22.5%, *p*-value ≤ 0.001), SFA 14.0 (mediation proportions: 67.17%, *p*-value ≤ 0.001), SFA 16.0 (mediation proportions: 175.86%, *p*-value ≤ 0.001),and SFA 18.0 (mediation proportions: 101.65%, *p*-value ≤ 0.001), as shown in Figure 7.

### 3.6 Subgroup analysis for the association between SFAs and depression

**Figure 8:**
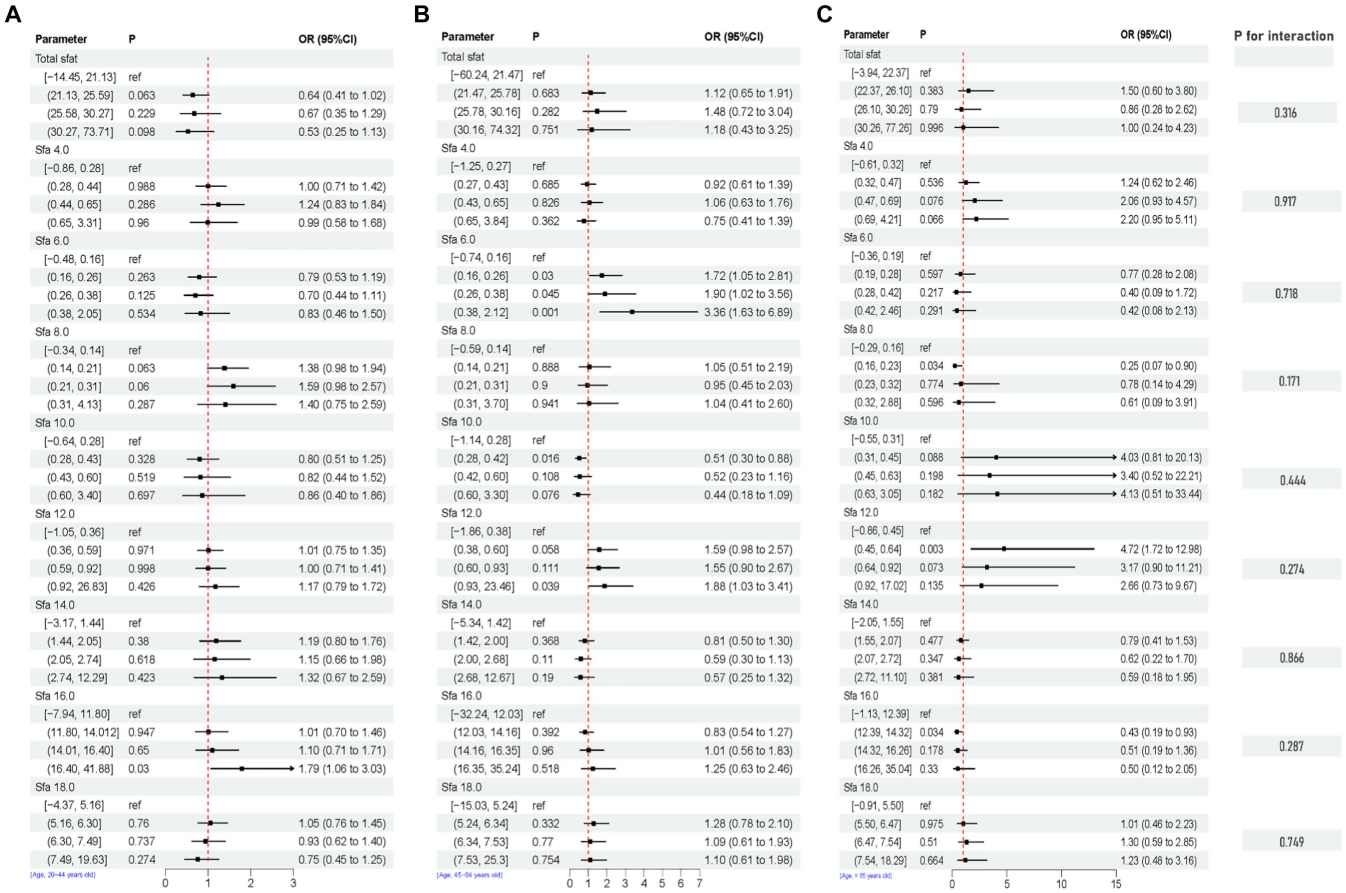
Multifactorial logistic regression of age differences and interaction of dietary saturated fatty acids and their subtypes with depression. Model adjusted for sex, age, ethnic/race, marital, family income-to-poverty ratio, education levels, PA, BMI, alcohol consumption status and smoke consumption status, DII. [**(A)** Age, 20–44, **(B)** Age, 45–64, **(C)** Age, ≥ 65].

**Figure 9:**
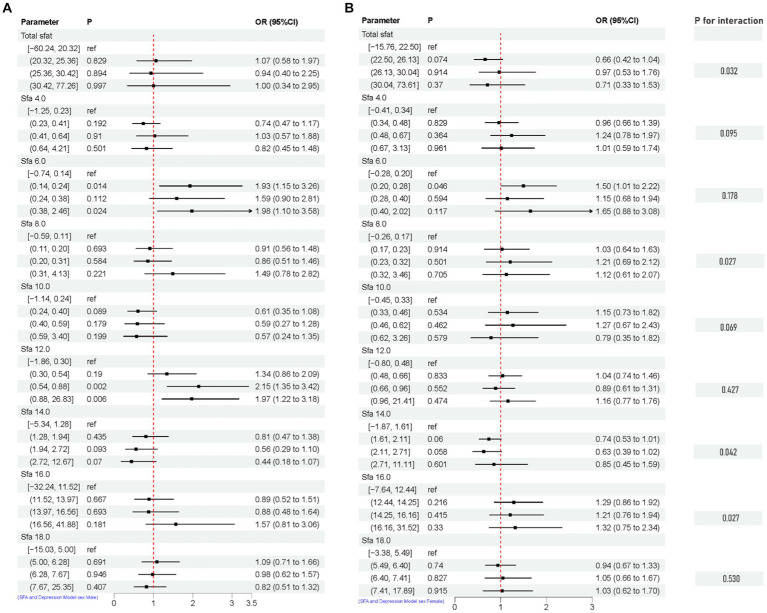
Multifactorial logistic regression of sex differences and interaction of dietary saturated fatty acids and their subtypes with depression. Model adjusted for sex, age, ethnic/race, marital, family income-to-poverty ratio, education levels, PA, BMI, alcohol consumption status and smoke consumption status, DII. [**(A)** Men, **(B)** Female].

After adjusting for multiple latent variables, the SFAs were all found to be significantly associated with the risk of prevalence of depression in different groups (regardless of sex and age). Among participants aged 20–44 years, SFA 16.0 [Q4, 1.79 (1.06, 3.03)] may be associated with an increased prevalence risk of depression. Among participants aged 45–64 years, SFA 6.0 [Q2, 1.72 (1.05, 2.81)], [Q3, 1.90 (1.02, 3.56)], [Q4, 3.36 (1.63, 6.89)], and SFA 12.0 [Q4, 1.88 (1.03, 3.41)] may be associated with an increased risk of developing depression. SFA 10.0 [Q2, 0.51 (0.30, 0.88)] may be associated with a decreased risk of developing depression. In participants (≥ 65 years old), SFA 8.0 [Q2, 0.25 (0.07, 0.90)], SFA 16.0 [Q2, 0.43 (0.19, 0.93)] may reduce the risk of developing depression. SFA 12.0 [Q2, 4.72 (1.72, 12.98)] may be associated with an increased risk of developing depression. SFAs did not have an interaction with age (*p*-value >0.05), as shown in Figure 8. Among male participants, SFA 6.0 [Q2, 1.93 (1.15, 3.26)], [Q4, 1.98 (1.10, 3.58)], SFA 12.0 [Q3, 2.15 (1.35, 3.42)], [Q4, 1.97 (1.22, 3.18)] may be associated with an increased risk of depression. SFA 14.0 [Q4, 0.44 (0.18, 1.07)] may be associated with a reduced risk of developing depression. In female participants, SFA 6.0 [Q2, 1.50 (1.01, 2.22)] may be associated with an increased risk of depression. Total sfat, SFA 8.0, SFA 14.0, SFA 16.0 interacted with sex (*p*-value ≤0.05), as shown in Figure 9.

## 4 Discussion

The study presents two significant new findings derived from the US general population. Firstly, SFA6.0, 8.0, and 12.0 were identified as risk factors for depression, while SFA10.0 and 14.0 were found to be protective factors. Furthermore, an age discrepancy was observed in the impact of SFA 16.0 on depression. Moreover, DII has been proposed as a mediator in the relationship between SFAs and the risk of depression.

Nutrition has been identified as a key factor in the prevention of depression, with a particular focus on dietary fats such as n-3 PUFA (6). While there is limited evidence on the role of SFAs, especially in relation to carbon chain length, studies have highlighted common sources of SFAs including coconut oil, rice bran oil, red meat, high-fat dairy products, and breast milk. Irregular dietary patterns can result in differing levels of SFAs intake, which have been associated with an increased risk of depression (6). Mechanistically, it has been reported that in a chronic unpredictable stress-induced rat depression model, SFA 6.0 plasma levels were higher in rats suffering from depression, and the relative abundance of *Anabaena spp.* was negatively correlated with SFA 6.0, while short-chain fatty acids have been suggested to be the key molecules in the regulation of depression (28, 29), and that Cistanche tubulosa extract was capable of reversing the SFA 6.0 in disordered concentrations to reasonable levels and may exert its antidepressant activity by altering the composition of the gut microbiota and SFAs levels (30). One study reported a potential association between SFA 8.0 and depression (31). Furthermore, a meta-analysis indicated that SFA8.0 could be a contributing factor to depression, possibly through the accumulation of medium-chain acylcarnitines leading to dysfunction in fatty acid metabolism and altered mitochondrial energy production. However, as direct evidence linking SFA 8.0 to depression is limited, additional validation in cohorts and more in-depth mechanistic studies are needed to further explore this relationship. Epidemiological evidence suggests that a high-fat diet, particularly one with approximately 60% of total energy from fat and a high SFAs content, can lead to depressive-like behaviors (32). Studies have shown that a high intake of SFAs may increase the risk of depressive symptoms in middle-aged women over a 4-years period (13). However, the findings regarding SFA 12.0 have been inconsistent. A randomized crossover study found no significant difference in depression scores among college students who consumed SFAs for a short period of 4 days (10). On the other hand, lauric acid has been associated with anti-inflammatory properties (33) and potential cognitive benefits, such as reducing inflammation in microglia and modulating cytokine and neurotrophic factor production (34, 35). A recent study using metabolomics identified elevated levels of SFA 12.0 in individuals taking psychiatric medications (36), suggesting a possible link between SFA 12.0 and depression. Further research in larger cohorts and basic experiments is needed to confirm the relationship between SFA 12.0 and depression.

SFA10.0, a fatty acid commonly found in medium-chain triglycerides in dietary fat, has been studied for its effects on spontaneous activity, anxiety-like and depression-like symptoms in male C57 BL/6 J mice (37). Research suggests that oral administration of SFA 10.0 may have a dose-dependent effect on reducing body weight and depression-related behaviors. Additionally, long-term administration of SFA 10.0 may exhibit antidepressant effects (37). SFA 10.0 easily penetrates the blood–brain barrier and enters the brain where it stimulates glycolysis and generates lactic acid within the mitochondria. The presence of lactic acid could potentially trigger panic-like reactions or exhibit anxiolytic and antidepressant properties (38–40). This may support our findings. Elevated levels of SFA 14.0 have been linked to depressive symptoms (6). Furthermore, SFA 14.0 levels in erythrocytes exhibited a negative correlation with the likelihood of developing depression (41). This may support our findings.

Our findings suggest that SFA 16.0 may be a risk factor for the risk of developing depression in those aged 20–44 years and a protective factor for the risk of developing depression in those ≥65 years. In addition, higher levels of SFA 16.0 were found to be associated with the risk of developing major depression in patients with major depression (42). Subjects with post-stroke depression in the elderly displayed elevated levels of SFA 16.0 in comparison to elderly individuals without depression (43). This contrasts with our own results, which could potentially be attributed to variances in outcomes stemming from dietary behaviors, regional disparities, and limited sample size. The study exclusively observed a rise in depression incidence within high SDI regions primarily among individuals (< 60 years old), while a decrease in depression rates was noted among older adults in these areas possibly due to enhanced social support and heightened focus on elderly care (44, 45). Furthermore, no direct association between SFA 16.0 levels and depression risk was identified (20–44 years old), emphasizing the necessity for larger-scale prospective cohort investigations and mechanistic analyses to elucidate the impact of SFA 16.0 on individuals with depression across different age brackets.

Our research discovered that SFAs can potentially increase the risk of depression by impacting levels of DII, but few studies in large cohorts have correlated and reported an intrinsic association. Two epidemiological studies indicate that higher consumption of SFAs is linked to elevated levels of hs-CRP (>3.0 mg/L), an inflammatory marker, and is positively associated in research involving adults from the US and Greece (46, 47). The DII serves as a tool for evaluating the combined impact of various pro- and anti-inflammatory components in the diet, with a meta-analysis revealing that lower DII scores were significantly linked to a reduced risk of depression (48). Furthermore, the suggestion that the connection between fatty acid intake and depression could be mediated by inflammatory markers (49) provides a theoretical foundation for our study, though the diverse subtypes of SFAs were not thoroughly considered. Moreover, the potential for a super-mediated effect in our findings may be due to the mediating variable encompassing not just the direct impact of SFAs on depression, but also the effect of other pathways or variables not captured by the model.

There are also some strengths to this study. First, it is a large sample study based on a complex sample. Second, the effect of differences in carbon chain length of saturated fatty acids on depression was fully considered. Finally, traditional statistical methods were used, with nutrients corrected and demographic da`ta weighted to reflect as much information as possible from the NHANES survey data. Since the study did not investigate the underlying mechanisms, variations in the results could stem from various causes. To begin with, internal mechanisms specific to SFAs and their subcategories, along with disparities in the confounding factors adjusted through regression analysis, may contribute to result disparities. Additionally, the utilization of the FFQ, a self-reported tool, could introduce inaccuracies in measuring SFA intake. Furthermore, the likelihood of reverse causation cannot be discounted in observational studies. Neyman bias might also impact the NHANES survey outcomes. Lastly, with only two dietary intake measurements averaged, the study could not capture long-term dietary patterns or changes following disease diagnosis.

## 5 Conclusion

Our study findings indicate that the consumption of specific SFAs may impact the prevalence of depression. Specifically, SFA 10.0 and 14.0 were associated with a decreased risk of depression, while SFA 6.0, 8.0, and 12.0 were linked to an increased risk. Furthermore, our mediation analyses suggest that the relationship between SFAs and depression risk may be influenced by dietary inflammation and the DII pathway. However, it is important to note that depression pathogenesis is multifaceted and other risk factors may also play a role. This study highlights the potential role of DII in mediating the negative effects of SFAs on depression.

## Data availability statement

The datasets presented in this study can be found in online repositories. The names of the repository/repositories and accession number(s) can be found below: all data entered into the analysis were from NHANES, which is publicly accessible to all (https://wwwn.cdc.gov/nchs/nhanes/Default.aspx).

## Ethics statement

The studies involving humans were approved by NCHS Ethics Review Board (ERB) Approval (https://www.cdc.gov/nchs/nhanes/irba98.htm). The studies were conducted in accordance with the local legislation and institutional requirements. Written informed consent for participation was not required from the participants or the participants’ legal guardians/next of kin in accordance with the national legislation and institutional requirements.

## Author contributions

CQ: Supervision, Writing – original draft, Writing – review & editing. RG: Data curation, Funding acquisition, Methodology, Supervision, Writing – original draft, Writing – review & editing.

## Footnotes

^1^ https://wwwn.cdc.gov/Nchs/Nhanes/2017-2018/DSQTOT_J.htm

^2^ https://wwwn.cdc.gov/Nchs/Nhanes/2003-2004/DR1IFF_C.htm#DR1IS040

^3^ https://cran.r-project.org/bin/windows/base/old/4.2.2/
