# Supplementary figures and images for: Association of dietary saturated fatty acid intake with depression: mediating effects of the dietary inflammation index
Source: Front Nutr. 2024 Jun 14;11:1396029. doi: 10.3389/fnut.2024.1396029 (PMC11211382; doi:10.3389/fnut.2024.1396029)

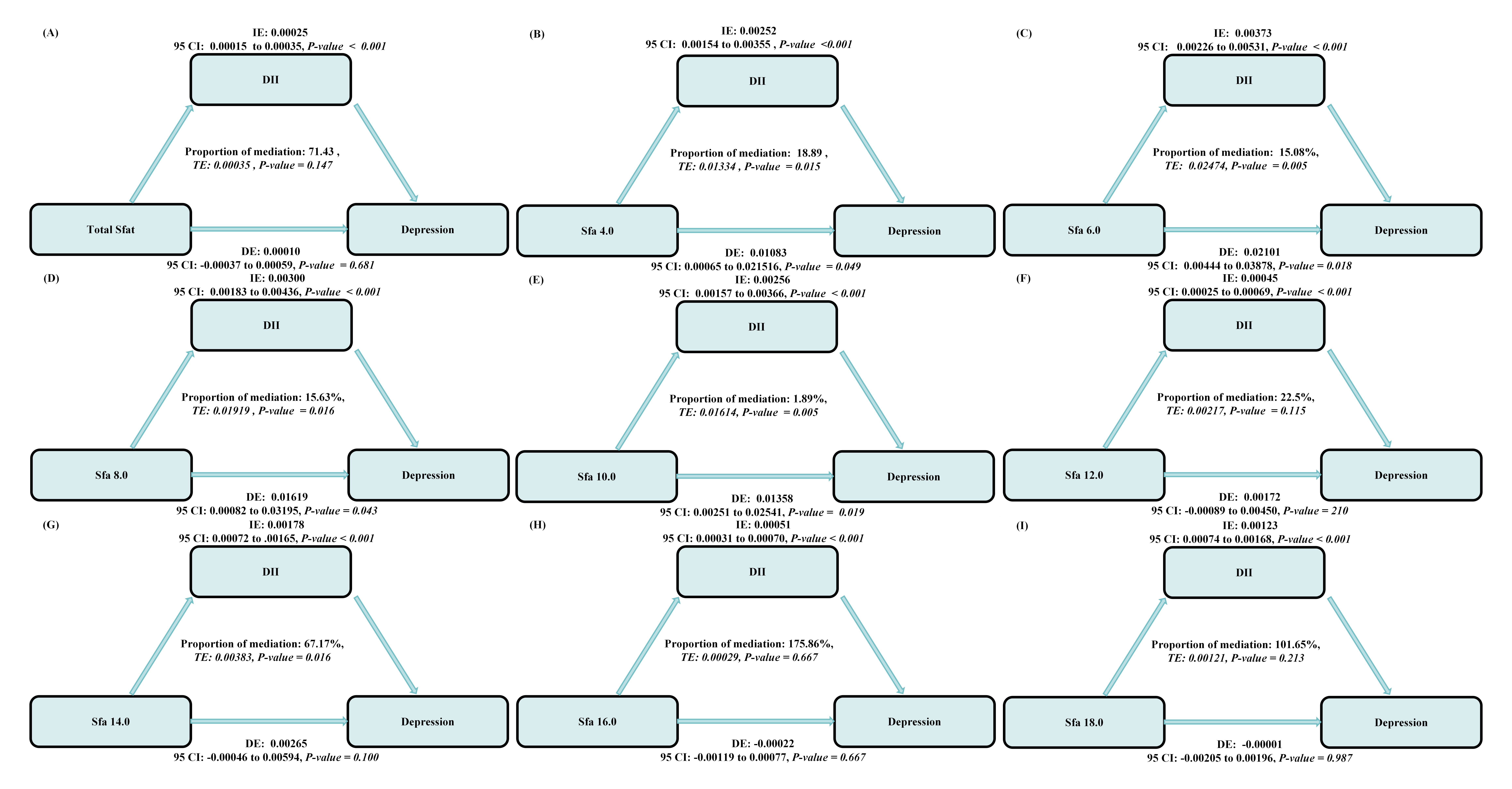

Supplement: Supplementary file 1 [file Image_1.PNG]

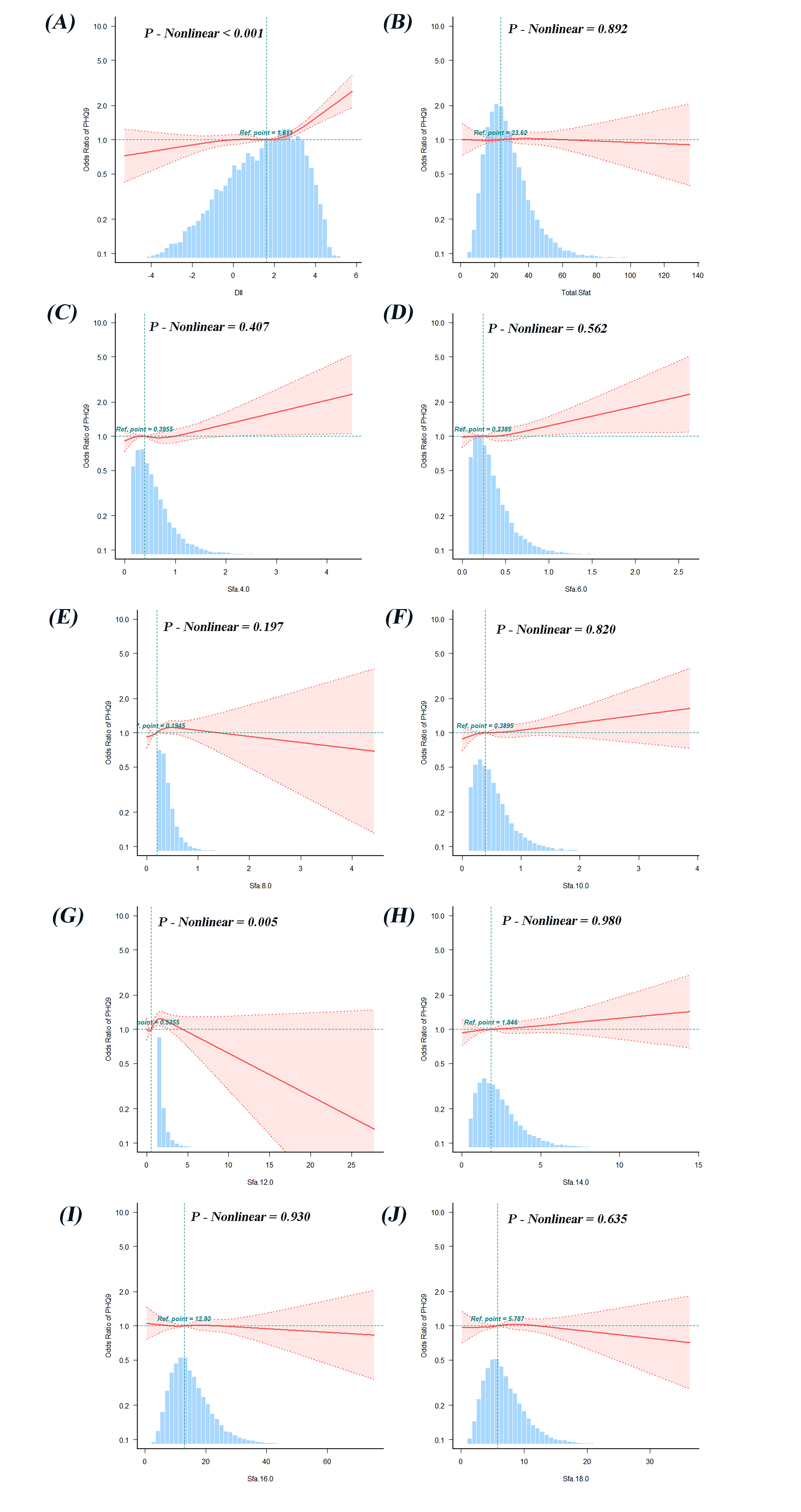

Supplement: Supplementary file 2 [file Image_2.PNG]

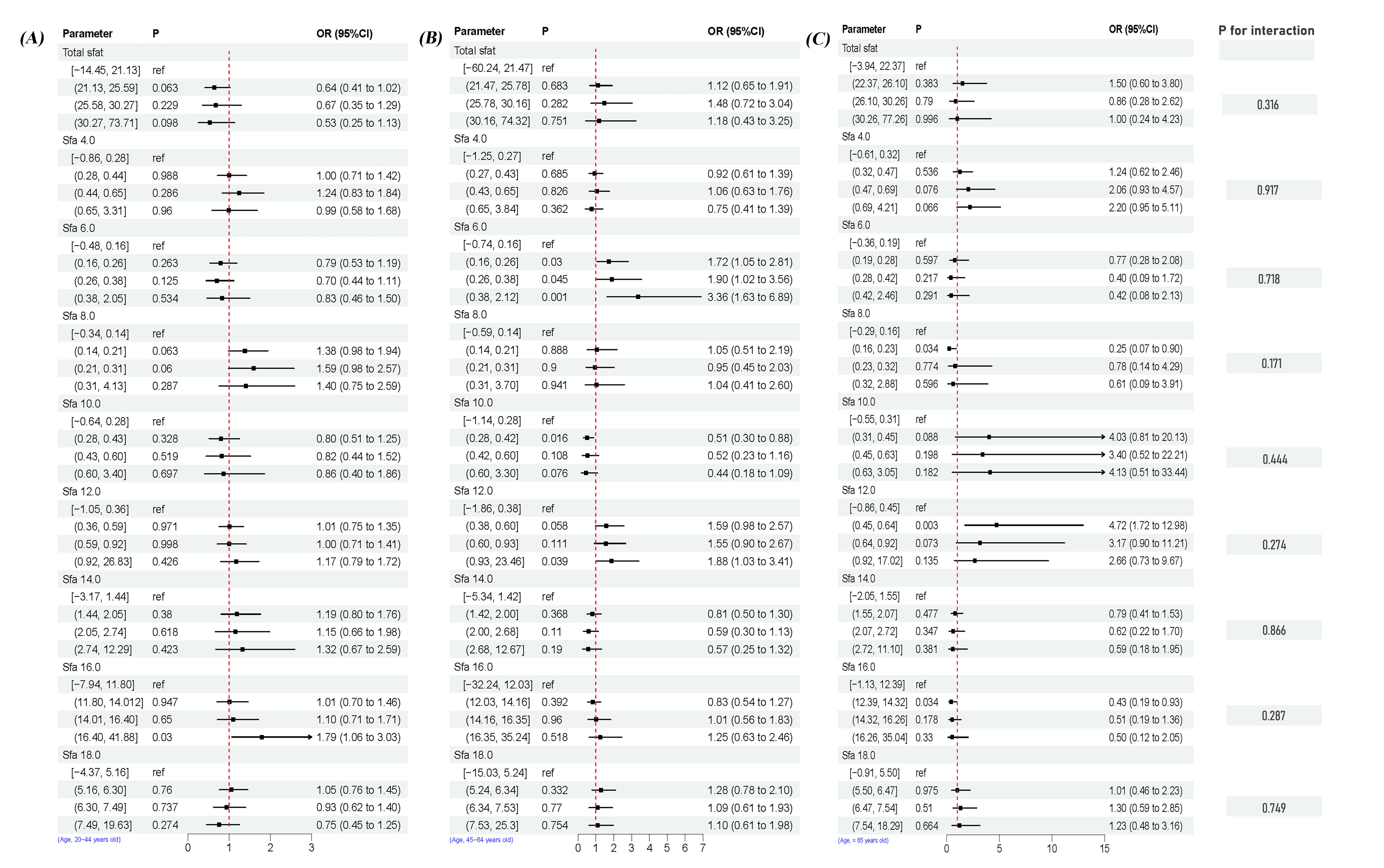

Supplement: Supplementary file 3 [file Image_3.PNG]

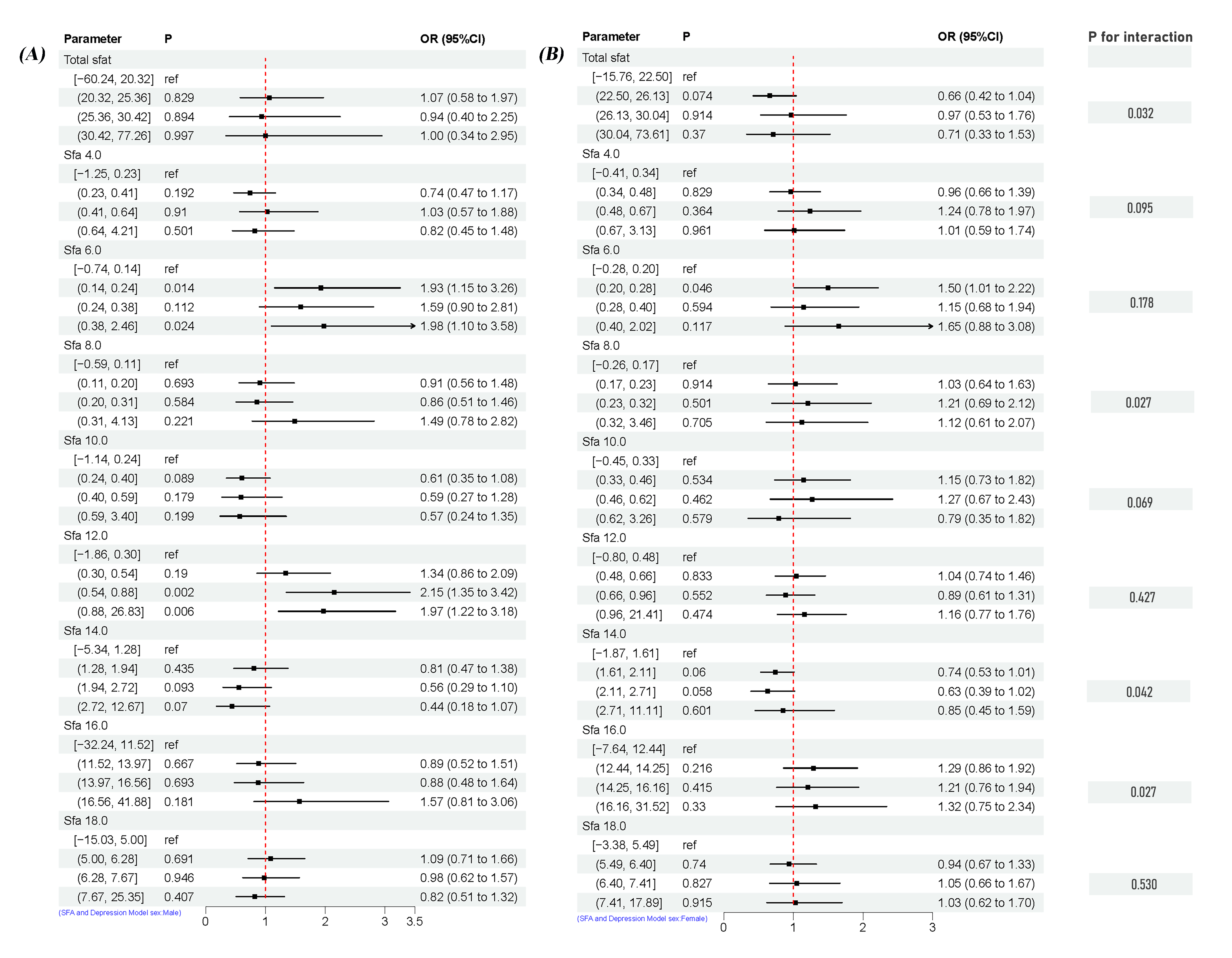

Supplement: Supplementary file 4 [file Image_4.PNG]

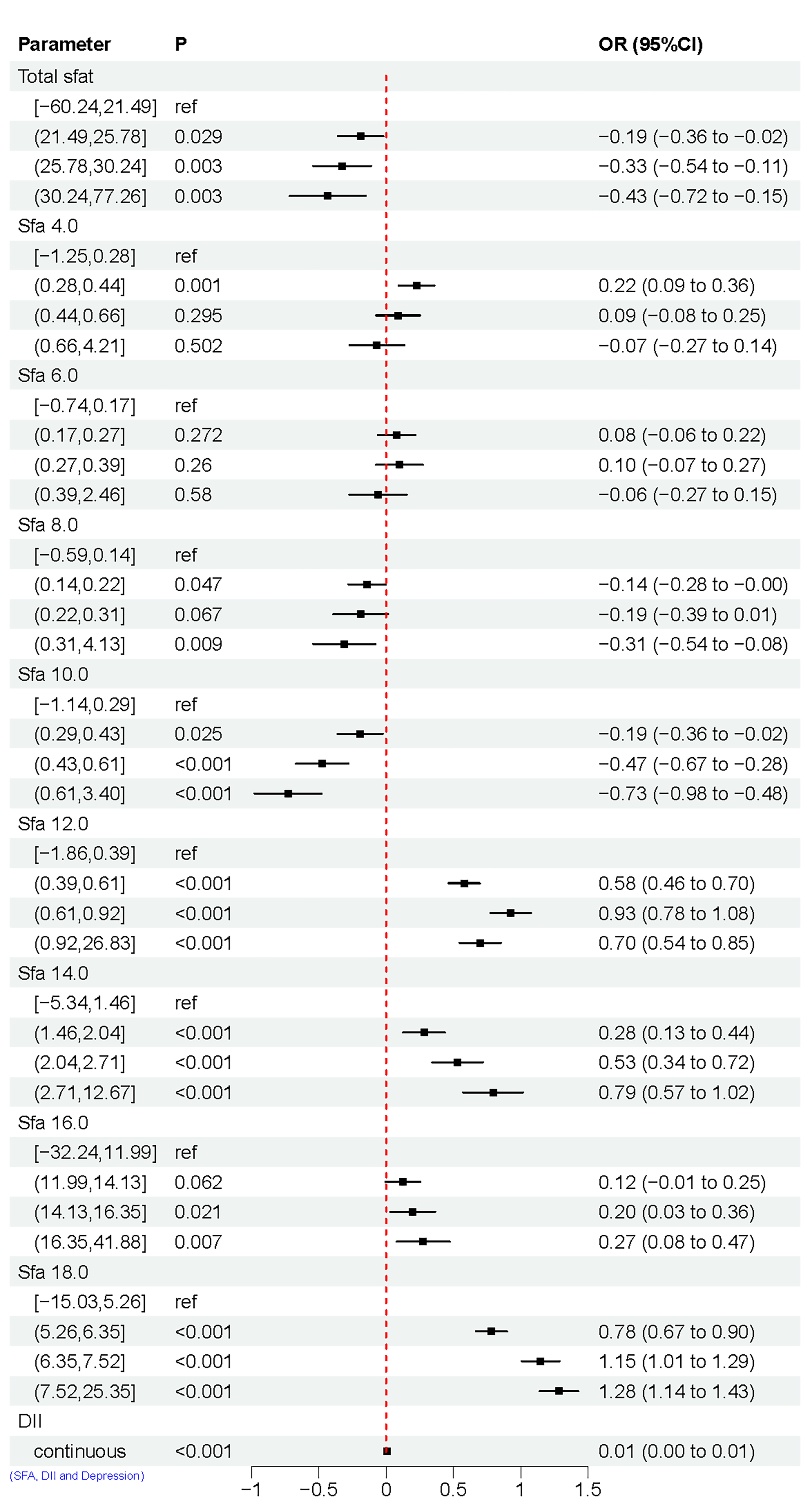

Supplement: Supplementary file 5 [file Image_5.TIF]

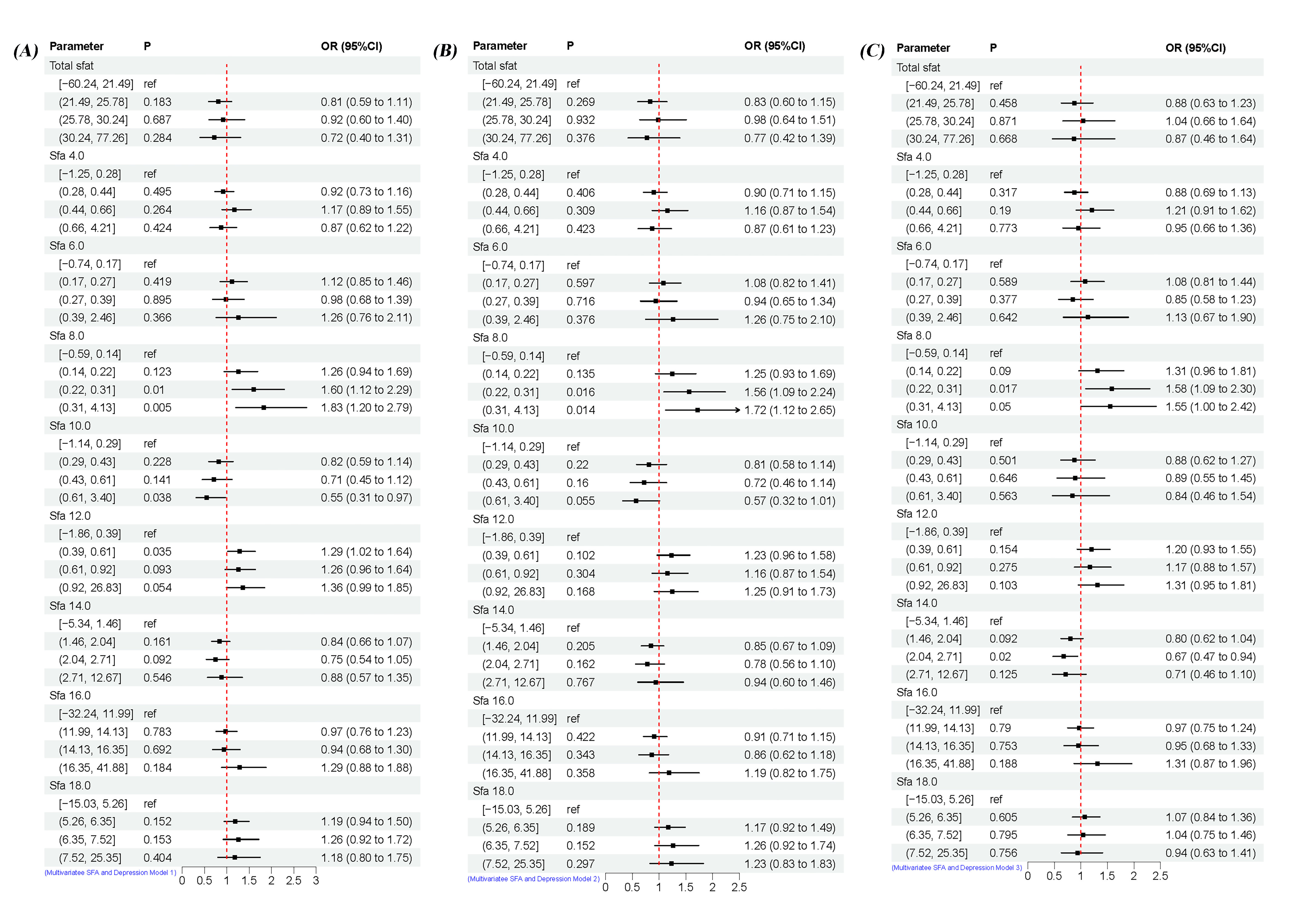

Supplement: Supplementary file 6 [file Image_6.PNG]

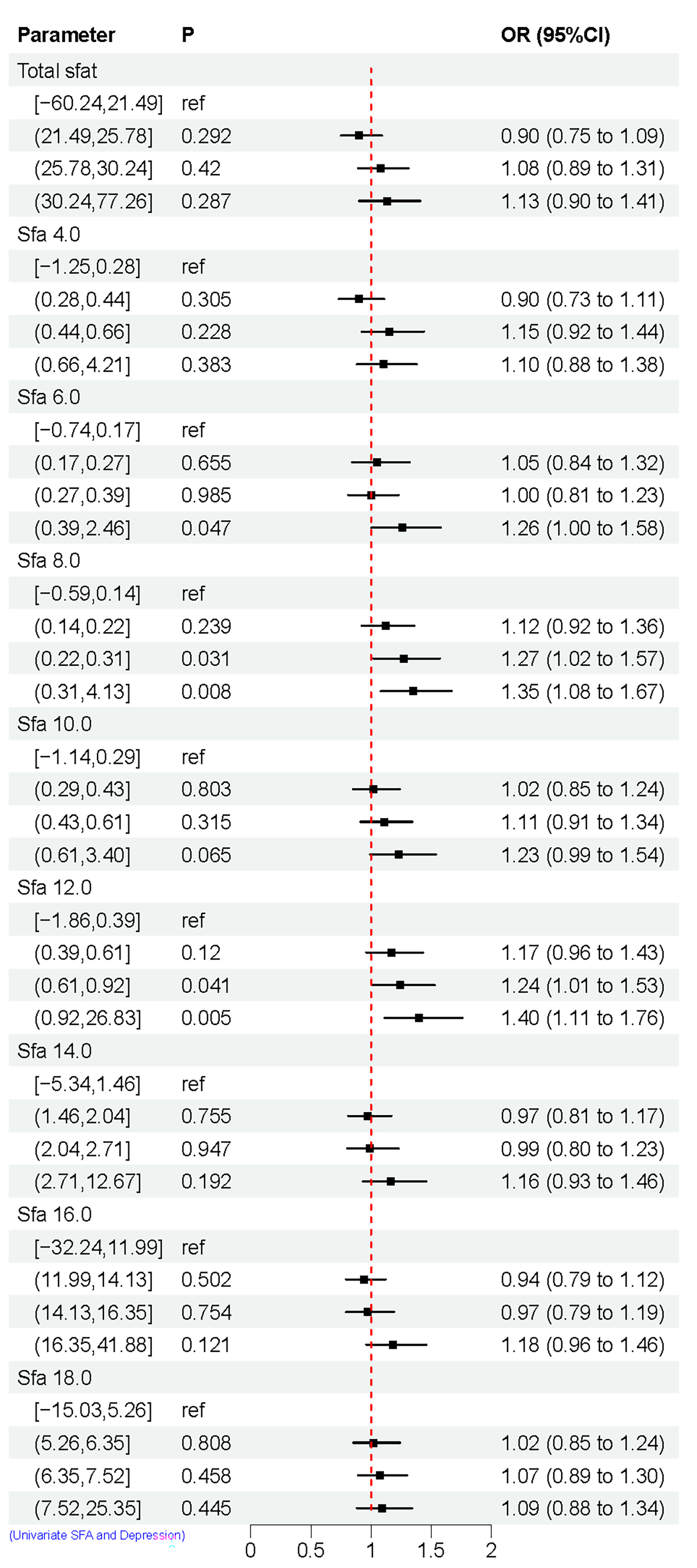

Supplement: Supplementary file 7 [file Image_7.PNG]
